# MicroRNAs in age-related diseases

**DOI:** 10.1002/emmm.201201986

**Published:** 2013-01-22

**Authors:** Stefanie Dimmeler, Pierluigi Nicotera

**Affiliations:** 1 Institute for Cardiovascular Regeneration, Centre of Molecular Medicine, Goethe University FrankfurtFrankfurt, Germany; 2 German Center for Cardiovascular Research (DZHK)Frankfurt, Germany; 3 German Center for Neurodegenerative Diseases (DZNE)Bonn, Germany

**Keywords:** cardiac, microRNA, neurodegeneration, senescence, vascular

## Abstract

Aging is a complex process that is linked to an increased incidence of major diseases such as cardiovascular and neurodegenerative disease, but also cancer and immune disorders. MicroRNAs (miRNAs) are small non-coding RNAs, which post-transcriptionally control gene expression by inhibiting translation or inducing degradation of targeted mRNAs. MiRNAs target up to hundreds of mRNAs, thereby modulating gene expression patterns. Many miRNAs appear to be dysregulated during cellular senescence, aging and disease. However, only few miRNAs have been so far linked to age-related changes in cellular and organ functions. The present article will discuss these findings, specifically focusing on the cardiovascular and neurological systems.

## Introduction

In recent times, the once unrealistic aspiration of long successful aging has been widely portrayed as a reachable target for both the individual and society. Unfortunately, the overall increasing age of the world population is accompanied by a rise in diseases including cardiovascular and neurodegenerative disease, cancer and immune disorders (North & Sinclair, 2012). The increase of the life expectancy in industrialized, but also developing countries results primarily from higher numbers of individuals aged above 65. For example, the number of Americans with an age above 65 is expected to double from 2000 to 2030 (Wang et al, 2010a) and in Europe, by 2050, there will be 48 million fewer 15–64 year-old people and 58 million more above 65 years of age. This undoubtedly will lead to an increase in age-associated diseases worldwide.

Despite the accumulating evidence that senescence is a major risk factor for human diseases, little is known about the molecular mechanisms that link the aging process to diseases. Research on aging has mostly been restricted to relatively short-lived organisms such as the small nematode *Caenorhabditis elegans*. With a normal life span of 21 days and the possibility to study short- or long-lived mutants, the worm has been an ideal model to identify genetic pathways that modulate aging. Significantly, most of these pathways are evolutionary conserved across species, including mammals. The insulin/insulin-like growth factor (IGF) signalling and the ‘target of rapamycin’ TOR signalling pathways have been studied in depth. Convergent findings show that inhibition of these pathways can extend longevity in model organisms (Kenyon, 2010). Also, major interventions that extend life span engage either or both of these pathways. For example, the TOR signalling cascade is involved in the extended life span due to dietary restriction, which appears to be universally associated with longevity in several organisms including mammals (Blagosklonny et al, 2009). Other relevant processes involved in aging include mitochondrial electron transport and respiration (Hughes & Hekimi, 2011; Moslehi et al, 2012).

A key component of the aging process is cellular senescence. Senescence does not occur simultaneously in all cells within a tissue, but involves irreversible proliferative arrest of damaged or dysfunctional cells (Rodier & Campisi, 2011). This contributes to restrict malignant progression of tumours, but also leads to accumulation of senescent cells within tissues. Senescent cells are not entirely quiescent, and recent evidence shows that they can actively promote aging and tissue damage (Baker et al, 2011). Targeted removal of senescent cells can delay the appearance of age-related phenotypes and organ dysfunction. Cell senescence is linked to complex changes in cellular morphology, structure and function (Rodier & Campisi, 2011). Major structural changes such as loss of the ends of the chromosomes (telomere erosion) accompany the irreversible growth arrest in senescent cells. Telomere erosion can be counteracted by the activity of telomerase, which is endogenously expressed in embryonic stem cells and cancer cells, and can reverse cellular aging in somatic cells when overexpressed (Boccardi & Herbig, 2012; Martinez & Blasco, 2011). Interestingly, telomere dysfunction has also been linked to metabolic changes suggesting an interplay between these two processes (Moslehi et al, 2012). Additional factors involve changes in protein processing, gene expression and miRNAs. As the factors leading to cell senescence become increasingly clear, it appears that dysfunction of the same pathways can occur in disease states. Several microRNAs (miRNAs) are involved in the regulation of pathways involved in cellular senescence and have effects on cell cycle progression. In addition, major changes in miRNA expression are involved in disease phenotypes in model organisms and have been found in tissues from human disease. The present article will discuss these findings, particularly focusing on the regulation and function of age-regulated miRNAs in cardiovascular and neurodegenerative diseases.

## MicroRNAs

MicroRNAs (miRNAs, miRs) are small, non-coding RNAs that post-transcriptionally control gene expression by blocking translation or inducing degradation of the targeted mRNA (for review see Bartel, 2009). In general, miRs are transcribed as primary transcripts by the RNA polymerase II. This primary transcript is further processed by the endonucleases Drosha and Dicer, resulting in the generation of a short RNA duplex. One strand of the duplex is loaded into the RNA-induced silencing complex (RISC) to bind to the target mRNA, whereas the other strand is usually (but not always) degraded. The regulation of miRNA biosynthesis and biochemical mode of action have been reviewed in detail elsewhere (*e.g.* see Ambros, 2004; Bartel, 2009; Bauersachs & Thum, 2011).

The small non-coding miRNA *lin-4* was first described in *Caenorhabditis elegans* as a regulatory gene essential for the transition through all the larval stages. During development, *lin-4* post-transcriptionally targets the nuclear factor LIN-14 and thus controls post-embryonic cell lineage patterns (Lee et al, 1993). Since then, almost 2000 miRNAs have been identified in mammals. MiRNAs play key roles in regulating development and tissue homeostasis, and de-regulation of miRNAs is implicated in the pathophysiology of various diseases such as cancer, cardiovascular, neurological and metabolic diseases (Bonauer et al, 2010; Gascon & Gao, 2012; Hébert & De Strooper, 2009; Iorio & Croce, 2012; Thum, 2012). Based on the identification of disease-regulated miRNAs, the targeting of miRNAs as a therapeutic approach was tested in experimental disease models, and particularly the inhibition of miRNAs by anti-sense ‘anti-miRs’ or ‘antagomirs’ was shown to improve disease states in mouse models (Thum, 2012; van Rooij et al, 2008). Importantly, locked nucleic acid inhibitors directed against miR-122 were successfully used to treat hepatitis in non-human primates in a Phase II clinical trial (NCT01200420; Lindow & Kauppinen, 2012), suggesting that modulation of miRNAs might be a therapeutic option. De-regulation of miRNAs has also been shown during cellular senescence and aging *in vivo*. We will summarize the current knowledge regarding the involvement of miRNAs in aging, with a focus on the regulation and function of age-regulated miRNAs in cardiovascular and neurodegenerative diseases.

## MiRNAs and aging

Aging is a biological process highly regulated by multiple evolutionary conserved mechanisms. Most of the involved signalling pathways converge in nutrient sensors that control intracellular metabolism and gene expression (Bano et al, 2011; Kenyon, 2010). *C. elegans* lifespan is regulated by Insulin/IGF-1 signalling (IIS; reviewed in Kenyon, 2010). DAF-2 (an insulin receptor-like protein) regulates the expression of the transcription factors abnormal dauer formation-16 (DAF-16) and heat shock factor-1 (HSF-1; Hsu et al, 2003). Loss of daf-2 function increases lifespan, while gain of daf-16 function is required for extended survival, which can be antagonized by daf-2 in wild-type *C. elegans* (Lin et al, 2001). More recent evidence has showed that miRNAs can contribute in the regulation of longevity. In *C. elegans*, loss-of-function of the heterochronic gene *lin-4* significantly shortens the lifespan, whereas *lin-4* overexpression extends the survival of nematodes. As demonstrated by epistasis analysis, this lifespan modulation requires the *lin-4* target gene *lin-14* (Boehm & Slack, 2005). Although it is still unclear by which mechanism the lin-4/lin-14 axis controls aging, these two temporal regulators of development genetically interact with some components of the Insulin/IGF-1 signalling pathways. In fact, *lin-14* loss-of-function is unable to affect the survival of the long-lived Insulin/IGF-1 deficient daf-2 (e1370) mutant, but it requires the downstream target DAF-16/FOXO to lengthen *C. elegans* lifespan. Although most miRNAs are dispensable for normal lifespan of model organisms, some other miRNAs can affect nematode longevity through DAF-16/FOXO-mediated gene transcription (de Lencastre et al, 2010). Among them, miR-71 remarkably coordinates longevity in a cell-non-autonomous manner. In wild-type *C. elegans*, miR-71 loss-of-function significantly decreases resistance to both heat shock and oxidative stress. Interestingly, miR-71 abolishes the lifespan extension of germline-less animals. Thus, along with secreted signals from the reproductive system, miR-71 likely targets unknown factors in the nervous system and, as a result, coordinates the nuclear redistribution of DAF-16/FOXO in the intestine, the main adipose tissue in nematodes (Boulias & Horvitz, 2012).

Meanwhile various miRNAs have been identified by microarrays or deep sequencing, which are regulated during *C. elegans* aging (Ibanez-Ventoso et al, 2006; Kato et al, 2011). Specifically, *let-7* is downregulated with advanced aging (Ibanez-Ventoso et al, 2006; Kato et al, 2011), which is consistent with the known function of *let-7* in the suppression of the steroid hormone receptor *daf-12* and therefore life span. Interestingly, in mice, *let-7* targets various components of the Insulin/IGF-1/mTOR pathway through the RNA-binding protein *Lin28* (Zhu et al, 2011), with important consequences for glucose metabolism. The muscle-enriched miR-1 also declined during aging (Ibanez-Ventoso et al, 2006). Upregulated miRNAs include miR-71, miR-238, miR-239 and miR-246 (de Lencastre et al, 2010). Whereas deletion of miR-71, miR-238 and miR-246 decreased life span, deletion of miR-239 had the opposite effect and extended life span (de Lencastre et al, 2010). Another age-induced miRNA, namely miR-34, did not affect *C. elegans* life span in one study (de Lencastre et al, 2010) but its inhibition enhanced life span by regulating autophagy in another study (Yang et al, 2011). In flies, loss of miR-34 triggered a decline in survival (Liu et al, 2012).

Several miRNA expression profiles have been generated to determine changes during aging in mammalian cells and tissues. While main endpoints of miRNA changes in *C. elegans* included life span and metabolic modifications, studies in mammalian systems have focused on specific tissues and systemic alterations. For example, a recent study in mouse models of senescence suggested that miR-29 targets type IV collagen genes (Takahashi et al, 2012). Type IV collagen is important for the maintenance of the structure of extracellular matrix. Increases in miR-29 in elderly mouse tissues were shown to decrease type IV collagen expression and weaken the basement membranes in elderly tissues.

In humans, comparisons of miRNA expression between peripheral blood mononuclear cells of young *versus* old individuals revealed the downregulation of various miRNAs, among which two – namely miR-24 and miR-221 – were validated in a cohort of older individuals (Noren Hooten et al, 2010). Other groups studied miRNA expression during culture-induced senescence and showed that members of the miR-17–92 cluster, namely miR-17, miR-19b, miR-20a and miR-106a are downregulated in several cell types (Hackl et al, 2010). In addition, miRNA profiles have been generated to compare aortas from young *versus* old mice (Boon et al, 2011), in senescent cultured human umbilical endothelial cells (Menghini et al, 2009), human and macaque prefrontal cortex samples during post-natal development and aging (Somel et al, 2010) as well as in neurodegenerative diseases (see below). Nevertheless, only few miRNAs so far have been directly linked to age-related changes in cellular and organ functions. However, many have been directly connected with disease states and it is yet unclear whether changes in miRNA profiles are mostly involved in the onset of pathological changes or rather mark the end stage of the senescence process resulting in organ aging and dysfunction.

## MiRNAs and age-related cardiovascular diseases

**Figure 1:**
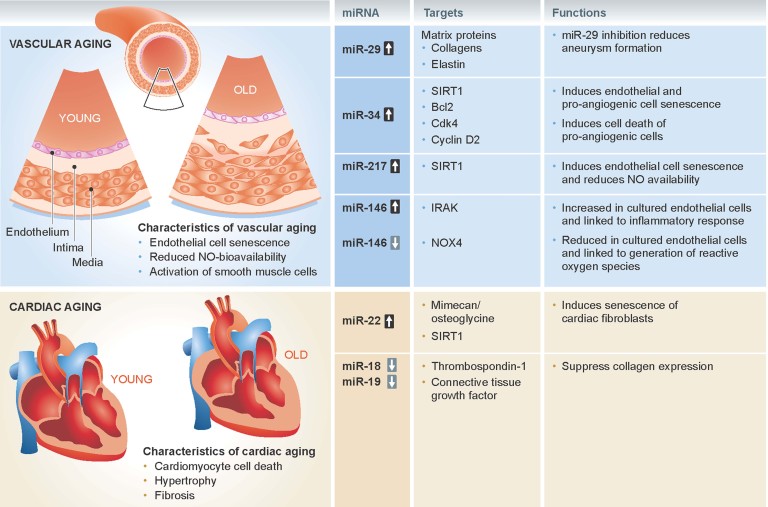
Regulation of miRNAs during cardiovascular aging The impact of aging on the vasculature and the heart is summarized. MiRNAs that are regulated during aging of the vasculature or the heart are shown with arrows indicating up- or downregulation during aging in comparison to young tissue. Only validated targets are shown.

Aging is the predominant risk factor for developing cardiovascular diseases (North & Sinclair, 2012). In the vasculature, aging impairs the atheroprotective effects of the endothelium as evidenced by reduced nitric oxide bioavailability and increased NFkB activation leading to endothelial inflammation (Pierce et al, 2009). Additionally, the smooth muscle cell phenotype is changed towards a pro-proliferative and pro-invasive characteristic and the composition of the extracellular matrix is significantly modulated (Wang et al, 2010a). Together, these changes result in arterial remodelling characterized by thickening of the intima and reduced arterial dilation capacity (Fig 1). It is considered that the changes in cellular functions and extracellular matrix during aging make the atrial wall susceptible for atherosclerotic lesion progression.

In the heart, the age-associated decline in cardioprotective systems additionally contributes to the development of heart failure (Strait & Lakatta, 2012). Thus, 90% of all heart failure deaths occur in individuals older than 70 (Strait & Lakatta, 2012). Aging is associated with an increase in fibrosis and contributes to diastolic dysfunction, which typically occurs in the elderly. In addition, aging contributes to a significantly worse outcome in patients with ischemic diseases such as acute myocardial infarction. The impaired neovascularization capacity during aging and the reduced function of vasculoprotective, proangiogenic cells and progenitor cells, which help to repair the vasculature and the heart after injury, may contribute to the age-associated worsening of recovery after critical ischemia (Dimmeler & Leri, 2008).

### MiRNAs in vascular aging

Several studies have addressed the regulation of miRNAs during culture-induced senescence of vascular cells (Hackl et al, 2010; Menghini et al, 2009; Olivieri et al, 2012) or in vessels that were isolated from aged mice (Boon et al, 2011). In cell culture studies, human umbilical venous endothelial cells were continuously cultured (population doubling time: 44) and miRNAs were detected in these senescent endothelial cells using microarrays. miR-21, miR-216, miR-217, miR-181b, miR-31b, miR-34a were among the confirmed upregulated miRs (Menghini et al, 2009), whereas members of the miR-17–92 cluster were downregulated (Hackl et al, 2010). In whole aortas of aged mice, the inflammation- and haematopoiesis-associated miR-146, miR-142-3p and miR-223 and the matrix-regulating miR-29 family were significantly increased, while other miRNAs, which were highly expressed in senescent endothelial cells, such as miR-181c/d and miR-31, were downregulated (Boon et al, 2011). These differences between the profiles of cultured endothelial cells and entire vessels likely reflect the different cellular compositions since in whole vessels smooth muscle cells and invaded inflammatory cells, but not endothelial cells are the predominant cell types. Therefore, whole tissue miRNA profiles have to be interpreted with caution, since the differential expression of miRNAs in selected cell types may be hidden behind the total changes in the tissue. Several of the regulated miRNAs have meanwhile been investigated in more detail (Fig 1).

#### miR-29 in age-associated vascular remodelling

In aortas from young *versus* old mice, members of the miR-29 family, namely miR-29a, miR-29b and miR-29c are upregulated (Boon et al, 2011). The age-induced increase in miR-29 family members is consistent with the upregulation of this miRNA family in several tissues of Zmpste24^−/−^ mice, a model for Hutchinson–Gilford progeria that exhibits accelerated aging and recapitulates many symptoms of normal aging (Ugalde et al, 2011). In addition, miR-29a was among the significantly increased miRNAs in cultured senescent endothelial cells (Menghini et al, 2009; Olivieri et al, 2012), and miR-29 was shown to contribute to senescence in cervical carcinoma cells (Martinez et al, 2011). The miR-29 family is well known to repress matrix proteins in the heart and liver and the increased expression of miR-29 family members in the aorta of aged mice was associated with a decline of matrix protein expression such as collagens and elastin (Boon et al, 2011). Since a reduction of extracellular matrix is considered to contribute to aneurysm formation, which is a typical age-associated vascular disease, several groups studied whether miR-29 inhibition could be exploited to improve the structural integrity of the vascular wall. Indeed, inhibition of miR-29 by anti-miRs enhanced matrix protein expression in Angiotensin II-induced aneurysms in aged mice as well as in genetic models of aneurysm formation (Boon et al, 2011; Maegdefessel et al, 2012; Merk et al, 2012; Zhang et al, 2012a). Of note, systemic inhibition of miR-29 may cause fibrosis or tumour growth, therefore warranting a local delivery strategy for putative therapeutic development.

The mechanism by which miR-29 is increased during aging is not entirely clear. Aging induces the primary miR-29b1-29a cluster (which encodes miR-29b and miR-29c), whereas the expression of the primary miR-29b2-29c cluster (encoding miR-29b and miR-29c) is not regulated in aged vessels (Boon et al, 2011). However, mature miR-29a, miR-29b and miR-29c were increased in the aorta of aged mice (Boon et al, 2011) and in Zmpste24-null mice, a mouse model of Hutchinson–Gilford progeria (Ugalde et al, 2011), indicating that age-induced upregulated involves transcriptional and post-transcriptional mechanisms (Boon & Dimmeler, 2011).

#### miR-34a in vascular aging

MiR-34a is not only increased during culture-induced endothelial cell senescence (Ito et al, 2010; Menghini et al, 2009; Olivieri et al, 2012) but also in different organs of aged mice (Ito et al, 2010; Li et al, 2011b). miR-34a expression was augmented in cultured endothelial progenitor cells and bone marrow-derived pro-angiogenic cells that were isolated from older patients with coronary artery disease or from aged mice (Tabuchi et al, 2012; Xu et al, 2012) and during *C. elegans* aging (de Lencastre et al, 2010) suggesting conserved age-induced expression of this miRNA. Overexpression of miR-34a induced senescence and inhibited proliferation of endothelial cells (Ito et al, 2010) and pro-angiogenic cultured endothelial progenitor cells (Zhao et al, 2010). MiR-34 is transactivated by p53 and promotes apoptosis (Chang et al, 2007). Indeed, miR-34a also induced cell death of bone marrow-derived proangiogenic cells (Xu et al, 2012), whereas inhibition of miR-34a reduced cell death of bone marrow-derived pro-angiogenic cells *in vitro* and augmented the capacity of the cells to improve cardiac function after acute myocardial infarction (Xu et al, 2012). In endothelial cells and endothelial progenitor cells, the class III histone deacetylase SIRT1 was shown to be the predominant target of miR-34a (Ito et al, 2010; Tabuchi et al, 2012; Zhao et al, 2010). SIRT1 is essential for endothelial cell functions and neovascularization after ischemia (Potente et al, 2007) and has been considered to reduce cellular senescence and enhance longevity (Maxwell et al, 2012). The downregulation of SIRT1 by miR-34a is in line with the detrimental effects of miR-34a on endothelial and progenitor cells functions. In addition, miR-34a inhibition enhanced the expression of the anti-apoptotic protein Bcl-2 and several cell cycle regulators such as Cdk4 and Cyclin D2 in freshly isolated bone marrow-derived proangiogenic cells (Xu et al, 2012).

The mechanism underlying the increase in miR-34a expression in aging has not been elucidated. It is known that p53 induces the expression of the transcripts encoding miR-34 family members and promotes miR-34a processing (Chang et al, 2007; He et al, 2007; Tarasov et al, 2007). But the expression of miR-34 can be increased independent of p53 (Christoffersen et al, 2010) and is also regulated by DNA methylation of CpG islands of the miR-34 promoters (Lodygin et al, 2008). However, DNA methylation of the promoter of miR-34a of pro-angiogenic cells was not changed during aging (Xu et al, 2012). Given the known involvement of p53 in age-associated pathways (Sahin & DePinho, 2012), one may speculate that p53 might mediate the age-induced upregulation of miR-34a.

#### miR-146 in endothelial cell senescence

miR-146 represents an inflammation-associated miRNA that was shown to be augmented in senescent fibroblasts (Bhaumik et al, 2009). miR-146 was profoundly increased in senescent cultured umbilical venous and different aortic endothelial cells and the increased expression was linked to the senescence-associated secretory phenotype (Olivieri et al, 2012). Thereby, an increase in senescence was associated with a decrease in IRAK, which controls the inflammatory response, whereas inhibition of miR-146 increased IRAK expression (Olivieri et al, 2012). While it has recently been confirmed that miR-146 targets IRAK1 in the heart, this resulted in protection against immune hyper-responsiveness during ischemia/reperfusion injury (Chassin et al, 2012). In addition, other groups reported that miR-146 was not regulated (Hackl et al, 2010) or was even significantly decreased in senescent umbilical venous endothelial cells (Vasa-Nicotera et al, 2011). In the latter study, miR-146a overexpression in endothelial cells reduced the NADPH oxidase NOX4, suggesting that reduced miR-146a expression might increase reactive oxygen species generation during endothelial cells senescence (Vasa-Nicotera et al, 2011). The reason for the discrepant findings showing either an increased or decreased expression of miR-146a during endothelial cell senescence are unclear, however, one may speculate that miR-146 may be upregulated as a feed-back mechanism during prolonged culture to suppress the senescence-associated secretory phenotype. *In vivo* experiments are now warranted to determine the relevance of the regulation of miR-146a during vascular aging.

#### miR-217 in endothelial senescence and atherosclerosis

miR-217 was identified as the most profoundly regulated miRNAs in senescent human umbilical endothelial cells (Menghini et al, 2009). Its overexpression significantly suppressed SIRT1, which plays a crucial role in endothelial cells and is also targeted by the age-induced miR-34a (see above). The upregulation of miR-217 was associated with a downregulation of SIRT1 and further increased eNOS acetylation and reduced eNOS protein expression *in vitro* (Menghini et al, 2009). Furthermore, miR-217 levels were inversely correlated with SIRT1 and eNOS expression in human atherosclerotic plaques (Menghini et al, 2009) suggesting that these pathways are functional under pathophysiological conditions.

#### Other senescence-associated vascular miRNAs

Some members of the miR-17–92 cluster including miR-17, miR-20a and miR-106a were significantly downregulated in senescent human umbilical endothelial cells (Hackl et al, 2010), whereas in another screen miR-17-5p expression was increased (Menghini et al, 2009). Since the members of the miR-17–92a family play distinct roles in endothelial cells, including pro-proliferative activity of miR-17/miR-20 but anti-angiogenic function of miR-17, miR-20 and miR-92a (Doebele et al, 2010) and were shown to reduce oncogene-induced senescence in tumour cells (Hong et al, 2010), further studies are required to elucidate the regulation and function of the individual family members during vascular aging and senescence. In addition, oxidative stress can induce miR-200c (Magenta et al, 2011; Olivieri et al, 2012) and its overexpression induces endothelial cell senescence *in vitro* (Magenta et al, 2011).

### MiRNAs in cardiac aging

In contrast to the vasculature, little is known regarding the regulation of miRNAs during cardiac aging. Although miRNA profiling of hearts from old *versus* young mice showed profound regulation of various miRNAs and miRNA clusters, only the upregulation of miR-21 has been validated in this study (Zhang et al, 2012b). miR-21 is known for its profibrotic activity (Thum et al, 2008), which may well fit in the context of cardiac aging, but the precise function during cardiac aging remains to be determined.

A second profile in functionally well-characterized aged mice revealed that miR-22 and miR-24 are profoundly induced during life span (Jazbutyte et al, 2012). The function of miR-22 has been further characterized in detail and loss-and gain of function studies confirmed that miR-22 induces senescence and promotes migration of cardiac fibroblasts (Jazbutyte et al, 2012). These findings are consistent with studies of others showing that miR-22 induces senescence in tumour cells (Xu et al, 2011). The effects of miR-22 in cardiac fibroblasts were partially mediated by the targeting of mimecan/osteoglycine (OGN), which is a secretory protein that was initially shown to control bone formation, but also is expressed in cardiac fibroblasts and smooth muscle cells (Jazbutyte et al, 2012). However, miR-22 additionally induces hypertrophy and may act via other known targets such as SIRT1.

Furthermore, van Almen et al. showed that members of the miR-17–92a cluster, specifically miR-18 and miR-19, were decreased in mouse strains, which are characterized by age-induced cardiac fibrosis and dysfunction (van Almen et al, 2011). The authors further confirmed that the previously identified miR-18 and miR-19 targets thrombospondin-1 and connective tissue growth factor are increased in heart failure-prone aged mice and the inverse correlation of the miRNAs and the targets was confirmed in human samples (van Almen et al, 2011). *In vitro* studies further explored the function of miR-18 and miR-19 and showed that both miRNAs suppress collagen expression in cardiac myocytes (van Almen et al, 2011). Although the mechanism by which these miRNAs regulate collagen expression has not been fully elucidated, these findings may provide a link between the downregulation of miR-18/miR-19 with age-induced fibrosis. Finally, miRNAs such as miR-133 can also modulate age-independent cardiac conditions, including hypertrophy (Care et al, 2007) and it may be interesting to study the impact of hypertrophy-regulation miRNAs in cardiac remodelling during aging.

## MiRNAs and age-related neurodegenerative diseases

Aging induces multiple cellular and functional changes in the brain that are partially compensated by adaptive neuroprotective and neurorestorative processes. Typical age-associated degenerative changes include abnormal and dysfunctional axons and neuritis, a decline in the neurotransmitter network, and presence of amyloid plaques and lipofusin around blood vessels. Some of these changes are analogous to changes observed in neurological diseases, with the notable exception of neuronal loss (Morrison & Hof, 1997). The burden of neurodegenerative disorders is progressively growing as the proportion of older people increases. According to the WHO, by 2040, neurodegenerative diseases are going to be the second leading cause of death after cardiovascular diseases. The prevalence of dementia cases will increase by 100% in developed countries and by up to 300% in newly industrialized countries (Ferri et al, 2005). Alzheimer's disease is the most prominent neurodegenerative disorder. However, an increasing number of people are diagnosed with mixed forms of dementia, vascular dementia and dementia with Lewy bodies. Notably a growing number of Parkinson's disease patients develop dementia as the disease spreads and progresses (Perez et al, 2012). Despite age being the most prominent risk factor for neurodegenerative diseases, the mechanisms and molecular targets involved are unknown. MiRNAs have been implicated both in the aging program and in disease of the nervous system. Moreover, it is becoming clear that miRNAs regulate physiological processes such as synaptic plasticity, learning and memory (Table 1).

**Table 1:** Regulation of miRNAs during neural aging and neurodegenerative age-associated diseases

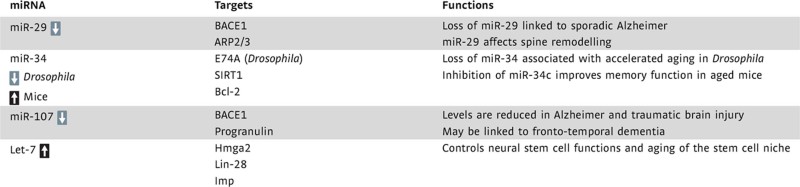

Profiling of tissue samples provided first evidence for regulation of miRNAs in the aging brain. In samples obtained from human prefrontal cortex, Somel et al. showed an age-associated upregulation of miR-34, miR-33b, miR-181 and miR-1271 (Somel et al, 2010). In macaque brain samples, similar patterns were observed including additionally miR-29b (Somel et al, 2010). In mice, age-associated increase in miR-30d, miR-34a, miR-468, miR-669b and miR-709 were observed in brain and liver tissue, whereas miR-22, miR-101a, miR-720, miR-721 were only increased in brain but not in livers of aged mice (Li et al, 2011a). Several of the increased miRNAs in the brain were predicted to control mitochondrial function, which is not surprising given that deregulation of mitochondrial function is a major factor in age-induced processes (Larsson, 2010). Maintenance of mitochondrial oxidative metabolism and energy production is particularly relevant at nerve endings, where mitochondria are essential to maintain protein turnover and exo-endocytotic processes at active synapses (Li et al, 2004). Long-term changes in synaptic strength also involve a rapid protein turnover. Therefore translational control at synapses is very important for structural plasticity. For example, the RISC protein MOV10 is rapidly degraded at synapses in an activity-dependent manner. This in turn causes several mRNAs to enter polysomes and drive protein expression (Banerjee et al, 2009). Evidence is accumulating that miRNAs have a significant role in modulating synaptic functional and structural plasticity (Lee et al, 2012; Lippi et al, 2011). It is therefore conceivable that altered local translational regulation may compromise learning and memory in dementia and other neurodegenerative disorders.

Initial evidence for the role of miRNAs in neurodegenerative diseases came from Schaefer et al (2007), who showed that conditional Purkinje cell-specific ablation of the key miRNA-generating enzyme Dicer resulted in cell death. Lack of *Dicer* was associated with progressive loss of miRNAs, cerebellar degeneration and development of ataxia *in vivo*. More recent findings confirm an important role for miRNA in age-associated neurodegeneration. Ablation of *Dicer* in adult forebrain caused abnormal tau hyperphosphorylation and neurodegeneration as observed in Alzheimer's disease brain. In view of these and other findings there are a number of studies of miRNA profiling in brain samples obtained from patients with neurodegenerative diseases. For example, miR-9, miR-125b and miR-146 are increased (Sethi & Lukiw, 2009) in temporal lobes neocortexes and hippocampal regions from Alzheimer's patients. Lukiw et al, also reported a significant upregulation of miR-9 and miR-128 compared to age-matched healthy adults (Lukiw, 2007). Various miRNAs are dysregulated in sporadic Alzheimer's disease patients according to a miRNA array (Hébert et al, 2008).

### miR-29 in Alzheimer's disease

Evidence that loss of the miR-29a/b-1 cluster is linked to sporadic Alzheimer's disease was provided by Hébert et al (2008). The study investigated possible regulators of the BACE1/β-secretase. BACE1 or APP may conceivably contribute to Aβ in sporadic Alzheimer's disease cases. BACE-1 processing of APP is essential to initiate Aβ generation, which is then promoted by the γ-secretase. MiRNAs that are potentially involved in the control of BACE1 expression (miR-29a, 29-b1) were found to be downregulated in brains of Alzheimer's patients that also showed high BACE1 levels. Along the same lines, these authors identified miR-20a, miR-17-5p and miR-106b as regulators of the Amyloid precursor protein (APP; Hébert et al, 2008, 2009).

Subsequent studies have found no genetic association of the genetic variants of the miR-29 clusters with Alzheimer's disease (Bettens et al, 2009). However, the lack of genetic variants does not rule out that miR-29 cluster downregulation modulates BACE-1 expression and APP processing. Also, the miR-29 cluster has other important roles in the brain that may be relevant to aging and disease. For example, expression levels of miR-29 affect dendritic spine remodelling by targeting the ARP2/3 actin nucleation complex. This is especially important for structural neuronal plasticity (Lippi et al, 2011). Interestingly, the decrease in miR-29 expression in diseased brains contrasts with the increase in miR-29 expression levels in other tissues during aging and the described role of miR-29 in mediating cellular senescence (see chapter 3).

### miR-34 in neurodegeneration

Levels of the miR-34 family change during aging in model organisms, mice and humans (Liu et al, 2012; Zovoilis et al, 2011). Loss of miR-34 is linked to accelerated aging and neurodegeneration in *Drosophila*. Interestingly, expression and processing of miR-34 varies with age in *Drosophila* and targets E74A. However, E74A has opposite effects on animal fitness during different life stages (known as antagonistic pleiotropy) and is not expressed in humans. Studies in mammalian models point at different regulatory roles for the different members of the miR-34 family. MiR-34c (but not miR-34a and miR-34b) is highly enriched in hippocampal regions and its expression is further upregulated by aging (24 months old mice), in mice models of amyloidosis (12 months APP/PS1-21 mice) and in Alzheimer's disease patients (Zovoilis et al, 2011). Targeting the miR-34 seed in APP/PS1-21 mice (were miR-34c is the predominant species) rescued learning dysfunction in mouse models (Zovoilis et al, 2011). One major target for miR-34c seems to be SIRT1 (Zovoilis et al, 2011). Decreased SIRT1 levels cause memory impairment and reducing elevated miR-34c levels in mice restored memory function in aged mice (Zovoilis et al, 2011). Given the multiple controls of autophagy and indirectly mitochondrial homeostasis, this seems a very promising target to achieve neuroprotection.

MiR-34a is upregulated in the cerebral cortex of other Alzheimer mouse models including the APPswe/PSΔE9 mice. Calorie restriction reduces miR-34a (and levels of miR-30e and miR-181a*) levels in hippocampus and cortex in aged mice. Decline of miRs was associated with increased Bcl-2 expression in aged mice after calorie restriction (Khanna et al, 2011). Notably, the p53-family member p73 controls the expression of miR-34a, which in turns targets mRNA encoding synaptic proteins (Agostini et al, 2011). This is relevant for neuronal development and plasticity. Given the limited evidence available we can only speculate that the miR-34 family controls several developmental and age-related metabolic pathways. Conceivably the targets of miR-34 are multiple and their functions may vary temporally and impact the aging process and disease.

### miR-107 in Alzheimer's disease

Expression of miR-107 decreases in Alzheimer's patients brains (Wang et al, 2008). Validation studies further confirmed that miR-107 levels are reduced in Alzheimer's disease brain neocortex (Nelson & Wang, 2010). As for miR-34c, miR-107 seems to regulate BACE1 levels (Wang et al, 2008). However, miR-107 has another relevant substrate linked to neurodegeneration: progranulin (Wang et al, 2010b). In traumatic brain injury a decreased level of miR-107 correlates with an increase in progranulin. Conversely, progranulin deficiency is linked to some forms of frontotemporal dementia (FTD), a major early onset age-dependent neurodegerative disease. Thus in FTD decreased levels of miR-107 may be beneficial to restore progranulin levels. Of note, recent findings indicate that miR-29b also negatively regulates progranulin levels (Jiao et al, 2010).

### Let-7 in neural stem cells

The Let-7 family has widespread effects on development and adult model organisms and mammalian cells. This is not surprising in view of recent findings that let-7 biogenesis is under multiple post-transcriptional controls including miRNA regulation of let-7 biogenesis (*i.e.* transcripts other than mRNA can be miRNA targets including pri-miRs such as pri-let-7; Zisoulis et al, 2012).

Let-7 was shown to control neural stem cell functions (Nishino et al, 2008; Tzatsos & Bardeesy, 2008) and affect cytokine secretion (Iliopoulos et al, 2009). Let-7 targets Hmga2, which promotes neural stem cell self-renewal in young but not old mice by reducing p16Ink4a and p19Arf expression (Nishino et al, 2008) and the stemness gene lin28. Let-7 has been shown to control aging of the Drosophila testis stem-cell niche and to mediate the age-dependent decrease in the IGF-II messenger RNA binding protein Imp leading to age-dependent decline in germline stem cells (Toledano et al, 2012). Premature senescence was also observed in fibroblasts after overexpression of let-7b, which downregulates EZH2 (Tzatsos et al, 2011). Another key target process for the let-7 miRNAs is metabolic regulation. Downregulation of Lin28a and Lin28b, or overexpression of let-7 in muscle causes insulin resistance and impaired glucose tolerance. The data presented in this study not only highlight the role of the Lin28a/b and let-7 as modulators of glucose metabolism, but also pose the question on how accumulation of let-7 in aging tissues may contribute to the systemic insulin resistance that accompanies aging. The role of let-7 in diseases like cancer is widely studied. However, evidence for a role in neurodegeneration is yet missing. In *C. elegans*, the amyloid precursor protein apl-1 (homologue to the human APP) is controlled by let-7 (Niwa et al, 2008), which is downregulated during development. A role in mammalian cells would be interesting in view of the self-modulation of let-7 activity.

## Conclusion and outlook

In conclusion, ample evidence suggests that miRNAs are deregulated during aging and some miRNAs have been implicated in age-associated decline of organ functions. Aging may induce general miRNA patterns that are similar for all organs. In contrast, miR-29 is differentially regulated and augmented in the aging vasculature or the heart, whereas it is suppressed in age-associated neurodegenerative diseases. Such an organ specific regulation is challenging when considering to therapeutically target miRNAs to prevent or ameliorate age-associated disease. For the potential development of therapeutic miRNA targeting strategies, it is furthermore of critical importance to understand whether the deregulated miRNAs during aging are causes or effectors of the age-associated disease or may even act as compensatory feed-back loops. The issue is further complicated by the understanding that miRNA are involved in more complex regulatory pathways in competition with other competitive endogenous RNAs (ceRNA) including pseudogenes and long non-coding RNAs (lncRNAs; Salmena et al, 2011). Given that such interactions may likely depend on the number of molecules of each interacting species, new models taking into account quantitative biology will be required to understand network modulation and finally develop therapeutic interventions. We believe that functional investigation of miRNAs and their interacting partners will open new perspectives for the understanding and the treatment of age-related disease processes.

**Pending issues**

Further identification and characterization of age-regulated miRNAs.

Establishment of functions and therapeutic strategies in age-related disease models.

Establishment of strategies to target miRNAs for chronic long term treatment – this likely requires cell-type specific delivery in case of miRNAs that have different functions in tissues/cells.

Understanding of the complex interactions with other competitive endogenous RNAs (ceRNA).

Conflict of interest statement: S.D. is member of the Scientific Advisory Board of Miragen and applied for patents regarding the use of miR-29 and miR-34.

**Glossary**

**Alzheimer's disease**

Age-related neurodegenerative disease, characterized pathologically by the accumulation of protein aggregates known as plaques and tangles consisting mainly of the proteins amyloid beta and phosphorylated tau. The main symptoms are memory loss and reduced cognitive function.

**Anti-miRs, antagomirs**

Backbone-modified antisense oligomers complementary to microRNAs, which block their function.

**Atherosclerosis**

Condition in which fatty material accumulates in the artery wall, which can restrict blood flow.

**Calorie restriction**

Reduced calorie intake diet with the aim to improve health and slow down the aging process.

**Frontotemporal dementia**

Early onset neurodegenerative disease caused by degeneration of the frontal and often the temporal lobes of the cerebral cortex. Symptoms vary depending on the brain regions affected.

**Hutchinson–Gilford progeria syndrome**

Rare genetic disease caused by progerin, a truncated version of the lamin A protein. Clinical manifestations include the appearance of aging beginning in childhood.

**Locked nucleic acid (LNA) technology**

Backbone modification of nucleic acids that locks the sugar moieties into a single constrained conformation, making the oligomers they are associated with nuclease-resistant.

**MicroRNA**

Small non-coding RNA molecule that controls gene expression by targeting messenger RNAs.

**Parkinson's disease**

Age-related neurodegenerative disease, characterized pathologically by the accumulation of protein aggregates in the brain, mainly composed of alpha-synuclein known as Lewy bodies. The main symptoms are reduced motor skills and movement defects.

**Reactive oxygen species (ROS)**

Chemically reactive oxygen-containing molecules. ROS levels increase in a cell during oxidative stress and cause damage to DNA, proteins and lipids.

**RNA-induced silencing complex (RISC)**

Ribonucleoprotein complex containing a small RNA such as a microRNA, which guides the RISC to complementary target messenger RNAs.

**Senescence**

The endogenous and hereditary process of accumulative changes in the passage of time resulting in functional deterioration and/or death.
